# An Electromyographic Analysis of Romanian, Step-Romanian, and Stiff-Leg Deadlift: Implication for Resistance Training

**DOI:** 10.3390/ijerph19031903

**Published:** 2022-02-08

**Authors:** Giuseppe Coratella, Gianpaolo Tornatore, Stefano Longo, Fabio Esposito, Emiliano Cè

**Affiliations:** 1 Department of Biomedical Sciences for Health, Università degli Studi di Milano, Via Giuseppe Colombo 71, 20133 Milano, Italy; gianpaolo.tornatore@email.it (G.T.); stefano.longo@unimi.it (S.L.); fabio.esposito@unimi.it (F.E.); emiliano.ce@unimi.it (E.C.); 2 IRCSS Galeazzi Orthopedic Institute, 20122 Milano, Italy

**Keywords:** hamstrings, gluteus, muscle activation, muscle excitation, electromyography, erector spinae, weight training, strength training

## Abstract

The present study examined the posterior chain muscle excitation in different deadlift variations. Ten competitive bodybuilders (training seniority of 10.6 ± 1.8 years) performed the Romanian (RD), Romanian standing on a step (step-RD), and stiff-leg deadlift (SD) with an 80% 1-RM. The excitation of the gluteus maximus, gluteus medius, biceps femoris, semitendinosus, erector spinae longissimus, and iliocostalis was assessed during both the ascending and descending phases. During the ascending phase, the RMS of the gluteus maximus was greater in the step-RD than in the RD (effect size (ES): 1.70, 0.55/2.84) and SD (ES: 1.18, 0.11/2.24). Moreover, a greater RMS was found in the SD than in the RD (ES: 0.99, 0.04/1.95). The RMS of the semitendinosus was greater in the step-RD than in the RD (ES: 0.82, 0.20/1.44) and SD (ES: 3.13, 1.67/4.59). Moreover, a greater RMS was found in the RD than in the SD (ES: 1.38, 0.29/2.48). The RMS of the longissimus was greater in the step-RD than in the RD (ES: 2.12, 0.89/3.34) and SD (ES: 3.28, 1.78/4.78). The descending phase had fewer differences between the exercises. No further differences between the exercises were found. The step-RD increased the overall excitation of the posterior chain muscles, possibly because of the greater range of movement and posterior muscle elongation during the anterior flexion. Moreover, the RD appeared to target the semitendinosus more than the SD, while the latter excited the gluteus maximus more.

## 1. Introduction

Resistance training is overall performed with the aim to increase muscle strength and induce structural adaptations [1,2]. While choosing the exercises to be included in a training routine, the technique of each constitutes the sum of the neuromuscular stimuli that muscles, tendons, and joints undergo. Therefore, appropriately selecting each exercise and its variations may target the warranted muscle structures.

Deadlift training is a multi-joint exercise that overall stimulates the gluteal, thigh, and back muscles and can be performed in a multitude of variations [3]. Basically, deadlifts can be divided into dynamic (e.g., regular or sumo deadlift) or isometric knee variations (e.g., Romanian or stiff-leg deadlift), where the knees perform a squat-like flexion/extension or maintain a constant angle during the whole exercise, respectively [3]. Particularly, the Romanian deadlift (RD) is performed with the knees slightly flexed, while the stiff-leg deadlift (SD) is performed with the knees extended. Although all deadlift variations strongly stimulate the targeted muscles, both the RD and SD are usually performed with the intent to enhance the stimuli to the posterior chain muscles [3]. This appears to derive from the lower role of the quadriceps, which are mainly involved in stabilizing the knees and much less involved in the dynamic lifting [4,5].

Both for the RD and SD, the movement starts and ends when the discs (or dumbbells or kettlebells) touch the ground. However, while the SD allows for a wide range of motion, this is limited when performing the RD. To overcome this problem, many practitioners perform the RD standing on a step (step-RD) to increase the displacement of the barbell. While both the RD and SD have received much attention [3], the step-RD has not been examined so far. Notwithstanding, a greater anterior flexion of the trunk would further elongate the posterior chain muscles, possibly increasing their excitation [6,7,8]. Additionally, bodybuilders have a unique capacity to perform exercises with profound consistency [9] and were recently involved in assessments of electromyographic activity in different exercises [7,8,10]. Importantly, when examining the muscle excitation [11] in each exercise, it is relevant to distinguish the ascending from the descending phase, which mostly corresponds to the concentric and eccentric actions of the targeted muscles since each phase show unique short-term [12], middle-term [13,14] and long-term adaptations when systematically performed [15,16].

Therefore, the present study aimed to investigate the posterior chain muscle excitation in competitive bodybuilders performing the RD, step-RD, and SD, during both the ascending and the descending phases. We hypothesized that the step-RD would result in greater muscle excitation due to the greater range of movement.

## 2. Materials and Methods

### 2.1. Study Design

The present investigation was designed as a cross-over, repeated-measures, within-subject study. The participants were involved in six different sessions. In the first session, the participants were familiarized with the technique of each exercise. In sessions two to four, the 1-RM was measured in the Romanian deadlift (RD), the step Romanian deadlift (step-RD), and the stiff-leg deadlift (SD). In the fifth session, the participants were familiarized with the selected loads and the electrode placements. In the sixth session, the muscles’ maximum excitation values were first measured. Then, after a minimum of 30 min of passive recovery, the participants performed a non-exhausting set for each exercise in a random order, with an inter-set pause of 10 min. Each session was separated by at least three days, and the participants were instructed to avoid any further form of resistance training for the entire duration of the investigation.

### 2.2. Participants

The present investigation was advertised by the investigators during some regional and national competitions, and to be included in the study, the participants had to compete in regional competitions for a minimum of 5 years. Additionally, they had to be clinically healthy, without any reported history of a lower limb or lower back muscle injury or neurological or cardiovascular disease in the previous 12 months. To avoid possible confounding factors, the participants competed in the same weight category (Men’s Classic Bodybuilding <80 kg, <1.70 m), according to the International Federation of Bodybuilding Pro League. The use of drugs or steroids was continuously monitored by a dedicated authority under its regulations, although we could not check for it. Thereafter, 10 male competitive bodybuilders (age of 29.8 ± 3.0 years; body mass of 77.9 ± 1.0 kg; stature of 1.68 ± 0.01 m; training seniority of 10.6 ± 1.8 years) were recruited for the present procedures. The participants were asked to abstain from alcohol, caffeine, or similar beverages in the 24 h preceding the test. After a full explanation of the aims of the study and the experimental procedures, the participants provided written informed consent. They were also free to withdraw at any time. The current design was approved by the Ethical Committee of the Università degli Studi di Milano (CE 27/17) and performed following the last Declaration of Helsinki for studies involving human subjects. The individual case presented in this manuscript gave written informed consent to publish these case details.

### 2.3. Exercise Techniques

**Figure 1:**
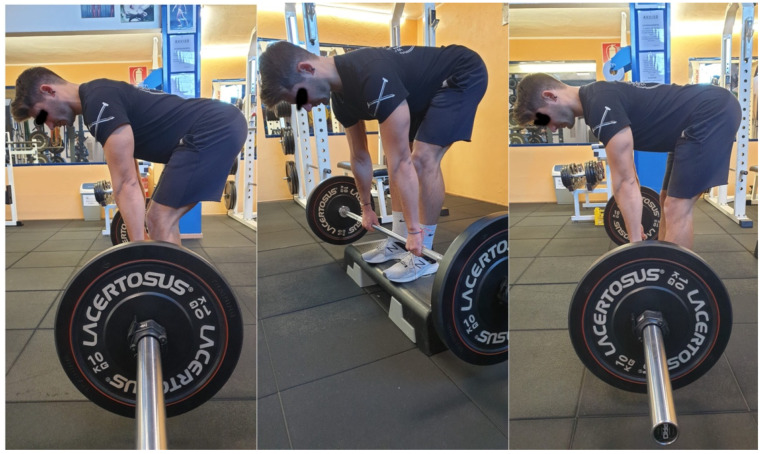
The technique for Romanian, step-Romanian and stiff-leg deadlift is shown.

The RD was performed standing with the feet hip-distance apart, with the knees slightly bent and a barbell placed in front of the participant (Olympic barbell Vulcan Standard 20 kg, Vulcan Strength Training System, Charlotte, NC, USA). The participants hinged forward at the hips, keeping the spine straight while reaching the trunk toward the floor. They gripped the barbell with both hands at shoulder-distance apart, drawing the shoulders back and down. Thereafter, they lifted the weight up to fully extend both the knees and hips. The step-RD was performed with the participant standing on a 15 cm step with the barbell on the floor. The SD was performed similarly but with extended knees for the whole movement. The technique of each exercise is shown in Figure 1. For each exercise, the time under tension was 2 s for the ascending and descending phases, with an isometric phase lasting approximately 0.5 s, and visual time feedback was provided [7,8,10,17]. After a warm-up consisting of 2 × 15 repetitions at a self-selected load, the participants performed six repetitions at an 80% 1-RM to avoid fatigue.

### 2.4. 1-RM Protocol

The 1-RM was assessed using the same exercise techniques as described above. Briefly, after a standardized warm-up consisting of 3 × 10 repetitions of the tested exercise using three incremental self-selected loads, the 1-RM attempts started from 80% of the self-declared 1-RM and an additional 5% or less was added until failure [1]. Each attempt was separated by at least 3 min of passive recovery. A metronome was used to pace the intended duty cycle. Strong standardized encouragements were provided to the participants to maximally perform each trial.

### 2.5. Maximum Voluntary Isometric Excitation

The maximal voluntary isometric excitation of the gluteus maximum, gluteus medius, biceps femoris, semitendineous, ileocostalis, and longissimus muscles was measured in random order following the surface electromyography for the non-invasive assessment of muscles (SENIAM) procedures [18]. The electrode (H124SG Kendall ARBO model; diameter of 10 mm; inter-electrodes distance of 20 mm; Kendall, Donau, Germany) placements were in line with the SENIAM recommendations [18]. The electrodes were equipped with a probe (probe mass of 8.5 g, BTS Inc., Milano, Italy) that permitted the detection and transfer of the surface electromyography (sEMG) signal by wireless modality. The sEMG signal was acquired at 1000 Hz, amplified (gain of 2000, impedance and the common rejection mode ratio of the equipment were >1015 Ω//0.2 pF and 60/10 Hz 92 dB, respectively) and driven to a wireless electromyographic system (FREEEMG 300, BTS Inc., Milano, Italy) that digitized (1000 Hz) and filtered (band-pass 10–500 Hz) the raw sEMG signals.

The sEMG electrodes for the gluteus maximus were placed at 50% on the line between the sacral vertebrae and the greater trochanter, with this position corresponding with the greatest prominence of the middle of the buttocks above the visible bulge of the greater trochanter [18]. The electrodes were orientated in the direction of the line from the posterior superior iliac spine to the middle of the posterior aspect of the thigh [18]. Starting in a prone position and lying down on a table, the participants were then instructed to lift the complete leg against manual resistance [18]. The electrodes for the gluteus medius were placed at 50% on the line from the crista iliaca to the trochanter in the direction of the line from the crista iliaca to the trochanter [18]. Starting in a prone position and lying down on a table, the participants were then instructed to press their legs against manual resistance while holding the ankles [18]. For the biceps femoris, the electrodes were placed at 50% on the line between the ischial tuberosity and the lateral epicondyle of the tibia, with an orientation in the direction of the line between the ischial tuberosity and the lateral epicondyle of the tibia [18]. The sEMG electrodes for semitendinosus were placed at 50% on the line between the ischial tuberosity and the medial epycondyle of the tibia, in the direction of the line between the ischial tuberosity and the medial epycondyle of the tibia [18]. For both the hamstring muscles, the participants laid face down with their thighs on the table and the knees flexed (to less than 90 degrees). The thighs were in a slight lateral rotation and the legs were in a slight lateral rotation with respect to the thighs. An operator pressed against the legs proximal to the ankles in the direction of the knee extension [18]. For the erector spinae longissimus, the electrodes needed to be placed laterally at a width of two fingers from the spinous process of L_1_, with a vertical orientation [18]. With the participants prone and their lumbar vertebral columns slightly flexed, they were instructed to lift their trunks. For the erector spinae ileocostalis, the electrodes were placed medially from the line at a width of one finger from the posterior spina iliaca superior to the lowest point of the lower rib, at the level of L_2_, in the direction of the line between the posterior spina iliaca superior and the lowest point of the lower rib [18]. Starting from a prone position with the lumbar vertebral columns slightly flexed, the participants were instructed to lift the trunk [18].

Each attempt lasted 5 s, and three attempts were completed for each movement, interspersed by 3 min of passive recovery [8,10]. The operators provided strong standardized verbal encouragement. In line with previous procedures, the electrodes were placed on the dominant limb [7,10,17].

To check for the appropriate electrode placements, the innervation zone shifts during movements for each muscle were checked by means of an 8 × 8 semi-disposable high-density electrodes matrix for sEMG detection (GR10MM0808 model, inter-electrode distance of 10 mm, OtBiolettronica Turin, Italy). The sEMG signal was acquired by a multichannel amplifier (EMG-USB model, OtBioelettronica, Turin, Italy; input impedance of >90 MΩ; CMRR of >96 dB; EMG bandwidth of 10–500; gain of 1000×) From the analysis of the sEMG signal, the innervation zone was identified and the muscle area involved in the innervation zone shift during the three exercises was avoided. Thereafter, the high-density electrode matrix was removed and replaced by the rounded electrodes.

### 2.6. Data Analysis

The sEMG signals from both the peak values recorded during the maximum voluntary isometric excitation and from the ascending and descending phases of each exercise were analyzed in a time domain using a 25-ms mobile window for the computation of the root mean square (RMS). For the maximum voluntary isometric excitation, the average of the RMS corresponding to the central 2 s was considered. During each exercise, the RMS was calculated and averaged over the 2 s of the ascending and descending phases. To identify the ascending and the descending phases, the sEMG was synchronized with an integrated camera (VixtaCam 30 Hz, BTS Inc., Milano, Italy) that provided the duration of each phase. Such a duration was used to mark the start and the end of each phase while analyzing the sEMG signal. The sEMG data were averaged, excluding the first and last repetition of each set to include possibly more consistent technique and decrease the interference of fatigue. After, the sEMG RMS of each muscle during each exercise was normalized for its respective maximum voluntary isometric excitation [7,8,10,17] and inserted into the data analysis.

### 2.7. Statistical Analysis

The statistical analysis was performed using statistical software (SPSS 27.0, IBM, Armonk, NY, USA). The normality of the data was checked using the Shapiro–Wilk test, and all distributions were normal (*p* > 0.05). Descriptive statistics are reported as the mean (SD). The differences in the normalized EMG RMS were separately calculated for the ascending and descending phases using a one-way repeated-measures ANOVA (factor exercise, 3 levels). Multiple comparisons were adjusted using Bonferroni’s correction. Significance was set at α < 0.05. The magnitude of the factor exercise was calculated using partial eta squared (η_p_^2^). The pairwise differences are reported as the mean Cohen’s d effect size (ES) with a 95% confidence interval (95% CI), and the ES was interpreted according to Hopkins’ recommendations, as follows: 0.00–0.19—trivial; 0.20–0.59—small; 0.60–1.19—moderate; 1.20–1.99—large; ≥2.00—very large [19].

## 3. Results

**Figure 2:**
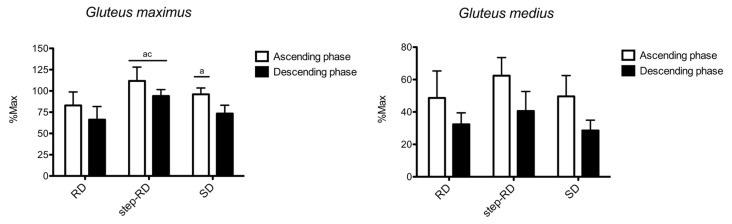
Normalized root means squares of gluteus maximus and gluteus medius shown for each exercise and phase. a = *p* < 0.05 vs. RD; c = *p* < 0.05 vs. SD.

Figure 2 shows the sEMG RMS recorded for the gluteus maximus and gluteus medius. For the gluteus maximus, the main effect for the factor exercise was found during the ascending (F_2,9_ = 6.549, *p* = 0.010, η_p_^2^ = 0.834) and descending phases (F_2,9_ = 14.094, *p* < 0.001, η_p_^2^ = 0.992). During the ascending phase, the RMS was greater in the step-RD than in the RD (ES: 1.70, 0.45 to 2.94) and SD (ES: 1.18, 0.11 to 2.24). Moreover, a greater RMS was found in the SD than in the RD (ES: 0.99, 0.04 to 1.95). During the descending phase, the RMS was greater in the step-RD than in the RD (ES: 2.16, 0.93 to 3.39) and SD (ES: 2.24, 0.99 to 3.49).

For the gluteus medius, no main effect for the factor exercise was found during the ascending (F_2,9_ = 2.894, *p* = 0.132, η_p_^2^ = 0.333) and descending phases (F_2,9_ = 3.765, *p* = 0.087, η_p_^2^ = 0.557). No differences between the exercises were found.

**Figure 3:**
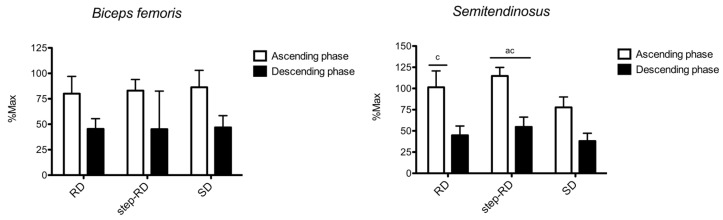
Normalized root means squares of biceps femoris and semitendinosus shown for each exercise and phase. a = *p* < 0.05 vs. RD; c = *p* < 0.05 vs. SD.

Figure 3 shows the sEMG RMS recorded for the biceps femoris and semitendinosus. For the biceps femoris, no main effect for the factor exercise was found during the ascending (F_2,9_ = 0.209, *p* = 0.817, η_p_^2^ = 0.065) and descending phases (F_2,9_ = 0.077, *p* = 0.927, η_p_^2^ = 0.025). No differences between the exercises were found.

For the semitendinosus, the main effect for the factor exercise was found during the ascending (F_2,9_ = 15.625, *p* = 0.004, η_p_^2^ = 0.839) and descending phases (F_2,9_ = 9.761, *p* = 0.013, η_p_^2^ = 0.765). During the ascending phase, the RMS was greater in the step-RD than in the RD (ES: 0.82, 0.20 to 1.44) and SD (ES: 3.13, 1.67 to 4.59). Moreover, a greater RMS was found in the RD than in the SD (ES: 1.38, 0.29 to 2.48). During the descending phase, the RMS was greater in the step-RD than in the RD (ES: 0.83, 0.20 to 1.45) and SD (ES: 1.50, 0.39 to 2.61).

**Figure 4:**
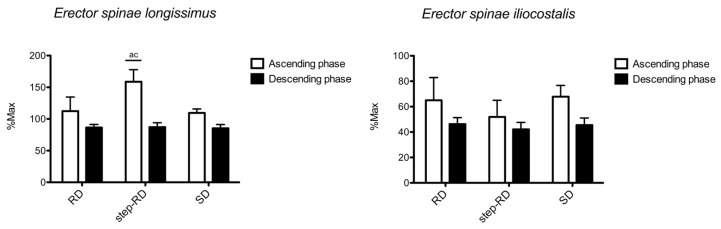
Normalized root means squares of erector spinae longissimus and iliocostalis shown for each exercise and phase. a = *p* < 0.05 vs. RD; c = *p* < 0.05 vs. SD.

Figure 4 shows the sEMG RMS recorded for the erector spinae longissimus and iliocostalis. For the erector spinae longissimus, the main effect for f the actor exercise was found during the ascending phase (F_2,9_ = 31.555, *p* < 0.001, η_p_^2^ = 0.912), but not during the descending phase (F_2,9_ = 0.195, *p* = 0.828, η_p_^2^ = 0.061). During the ascending phase, the RMS was greater in the step-RD than in the RD (ES: 2.12, 0.89 to 3.34) and SD (ES: 3.28, 1.78 to 4.78). No differences between the exercises were found during the descending phase.

For the erector spinae iliocostalis, no main effect for the factor exercise was found during the ascending (F_2,9_ = 2.754, *p* = 0.098, η_p_^2^ = 0.282) and descending phases (F_2,9_ = 3.663, *p* = 0.053, η_p_^2^ = 0.344). No differences between the exercises were found.

## 4. Discussion

The current study investigated the excitation of the main gluteal, rear thigh, and back muscles in three deadlift variations—the RD, step-RD, and SD. The observations during the ascending phase highlighted that the gluteus maximus, semitendinosus, and erector spinae longissimus were more excited in the step-RD than in both the RD and SD. Moreover, the gluteus maximus was more excited in the SD than in the RD, while the semitendinosus was more excited in the RD than in the SD. No differences were observed for the gluteus medius, biceps femoris, and iliocostalis. The descending phase followed a similar pattern, albeit fewer differences between the exercises were observed. The use of a step to perform the RD seemed to increase the muscle excitation of the gluteus maximus, semitendinosus, and longissimus compared to the RD and SD.

The gluteus maximus is located superficially to the gluteal region and, among other actions, is responsible for the hip extension [20]. Therefore, since the SD does not have any dynamic action coming from the knee extension, the barbell is mostly controlled by the hip flexion/extension. The literature reports that gluteus maximus RMS values increase with the external load [21,22], so the present data are in line with the literature. Notwithstanding, while the greater excitation in the SD than in the RD was expected, and although both have high RMS values, performing the step-RD appeared to increase the excitation of the gluteus maximus. This may be due to the greater displacement of the barbell below the feet, further elongating the posterior muscle chain. Previous studies have reported greater muscle excitation when performing a movement, leading to greater muscle elongation, including for the triceps brachii [6], different heads of the deltoid [8], and the adductor longus [7]. Interestingly, the RD appeared to increase the excitation of the semitendinosus more than the SD, while no difference was observed for the biceps femoris. In addition, it was previously observed that hip-dominant movements elicited more excitation in the biceps femoris than knee movements, the latter of which seemed to excite the semitendinosus more [23]. In the first instance, this may partially explain the greater excitation of the semitendinosus in the RD than in the SD. Additionally, although the greater elongation occurred in the step-RD, the SD may have compensated for the enhancement of the excitation of the biceps femoris given its hip dominance. The present data also highlight greater excitation of the semitendinosus than the biceps femoris, as has also been reported in previous studies [24,25].

The erector spinae muscles are crucial for controlling the load during any deadlift variation [5,24,25,26], as summarized earlier in a review [3]. The greater muscle elongation reached during the step-RD can also justify the greater excitation of the erector spinae longissimus in the step-RD compared to both the RD and SD, as shown previously [6,7,8]. In contrast, the iliocostalis showed no differences between the exercises and was much less excited than the longissimus. The iliocostalis acts on the spine at a shorter level compared to the longissimus, so its role is minor, as was also observed in different squat variations performed with a similar load (80% 1-RM) [7]. Lastly, the gluteus medius acts as a stabilizer of the hips, controlling both the abduction and the internal/external rotation. Since all exercises were mostly stable on the both frontal and transverse planes, such a lack of difference appears to be justified.

While it is known that the descending phase implies overall eccentric actions that require less muscle excitation than concentric actions for a given load [27], the differences between the exercises during the ascending phase were not exactly the same during the descending phase. For example, no differences between the exercises were observed for the erector spinae longissimus during the descending phase, possibly implying constant movement control when performing any deadlift variation. Additionally, no difference in the gluteus maximus and semitendinosus excitation was found between the RD and SD during the descending phase. However, differences still persisted when performing the step-RD compared to the RD and SD, possibly due to the greater range of movement, the greater elongation, and also a possible less stable position when standing on a step vs. the ground.

Some limitations accompany the present investigation. First, the data refer to the current population, so it is possible that different populations would have different muscle excitation results. Second, we focused on the posterior chain muscles, and further relevant muscles, such as the quadriceps, were not assessed. Third, we only examined three deadlift variations, and we acknowledge that a more comprehensive evaluation would require further exercises. Lastly, adding kinematic data would deepen the knowledge and should be considered in future research.

## 5. Conclusions

The results of the present investigation show that performing the step-RD increased the excitation of the posterior chain muscles compared to both the RD and SD. Moreover, while the RD appeared to increase the role of the semitendinosus, the SD resulted in a greater excitation of the gluteus maximus. In resistance training, the present outcomes may help practitioners to select deadlift variations depending on the aim of the session. Particularly, the use of a step appears to increase the range of movement, further elongate the posterior muscles, and increase their excitation.

## Data Availability

Data are available upon request from the corresponding author.
